# The value of muscle biopsies in Pompe disease: identifying lipofuscin inclusions in juvenile- and adult-onset patients

**DOI:** 10.1186/2051-5960-2-2

**Published:** 2014-01-02

**Authors:** Erin J Feeney, Stephanie Austin, Yin-Hsiu Chien, Hanna Mandel, Benedikt Schoser, Sean Prater, Wuh-Liang Hwu, Evelyn Ralston, Priya S Kishnani, Nina Raben

**Affiliations:** 1 Laboratory of Muscle Stem Cells and Gene Regulation, National Institute of Arthritis and Musculoskeletal and Skin Diseases, National Institutes of Health, Bethesda, MD 20892, USA; 2 Division of Medical Genetics, Department of Pediatrics, Duke University Medical Center, Durham, NC 27710, USA; 3 Department of Pediatrics and Medical Genetics, National Taiwan University Hospital and National Taiwan University School of Medicine, Taipei, Taiwan; 4 Metabolic Unit, Meyer Children’s Hospital, Rambam Medical Center, Technion Faculty of Medicine, Haifa, Israel; 5 Friedrich-Baur-Institute, Department Of Neurology, Ludwig-Maximilians University, Munich, Germany; 6 Light Imaging Section, Office of Science and Technology, National institute of arthritis and musculoskeletal and skin diseases, national institutes of health, Bethesda, MD, USA

**Keywords:** Pompe disease, Acid alpha-glucosidase, Skeletal muscle, Pathology, Autophagy, Lipofuscin, Muscle biopsy

## Abstract

**Background:**

Pompe disease, an inherited deficiency of lysosomal acid alpha-glucosidase (GAA), is a metabolic myopathy with heterogeneous clinical presentations. Late-onset Pompe disease (LOPD) is a debilitating progressive muscle disorder that can occur anytime from early childhood to late adulthood. Enzyme replacement therapy (ERT) with recombinant human GAA is currently available for Pompe patients. Although ERT shows some benefits, the reversal of skeletal muscle pathology - lysosomal glycogen accumulation and autophagic buildup - remains a challenge. In this study, we examined the clinical status and muscle pathology of 22 LOPD patients and one atypical infantile patient on ERT to understand the reasons for muscle resistance to ERT.

**Results:**

The patients were divided into three groups for analysis, based on the age of onset and diagnosis: adult-onset patients, juvenile-onset patients, and those identified through newborn screening (NBS). The areas of autophagic buildup found in patients’ biopsies of all three groups, contained large autofluorescent inclusions which we show are made of lipofuscin, an indigestible intralysosomal material typically associated with ageing. These inclusions, analysed by staining, spectral analysis, time-resolved Fluorescence Lifetime Imaging (FLIM), and Second Harmonic Generation (SHG) imaging, were the major pathology remaining in many fibers after ERT. The best outcome of ERT both clinically and morphologically was observed in the NBS patients.

**Conclusions:**

The muscle biopsy, in spite of its shortcomings, allowed us to recognize an underreported, ERT-resistant pathology in LOPD; numerous lysosomes and autolysosomes loaded with lipofuscin appear to be a hallmark of LOPD skeletal muscle. Lipofuscin accumulation - a result of inefficient lysosomal degradation - may in turn exacerbate both lysosomal and autophagic abnormalities.

## Background

Pompe disease (glycogen storage disease type II; OMIM 232300) is an inherited neuromuscular disorder caused by a deficiency of acid alpha-glucosidase (GAA; OMIM 606800), the sole enzyme responsible for the breakdown of glycogen in the lysosomal compartment [1,2]. Accumulation of undigested glycogen within lysosomes occurs in many tissues but is particularly detrimental to cardiac, skeletal, and smooth muscle. The complete or nearly complete absence of the enzyme results in the most severe infantile form of the disease, characterized by hypertrophic cardiomyopathy and skeletal muscle myopathy, onset soon after birth, and death from cardiorespiratory failure before one year of age. Partial enzyme activity, allowing for the protection of cardiac muscle, leads to progressive skeletal muscle myopathy; largely dependent upon levels of residual GAA activity, this form of the disease - known as late-onset Pompe disease (LOPD) - affects individuals with different degrees of severity and at varying ages of onset [3-5].

The natural history of the disease changed following the implementation of enzyme replacement therapy (ERT) with recombinant human GAA (rhGAA; alglucosidase alfa; Myozyme® and Lumizyme®; Genzyme Corp., Cambridge, MA). Rescuing cardiac muscle (and having a limited effect in skeletal muscle), the drug enables much longer survival of infantile-onset patients [6-8] but leaves them with skeletal muscle myopathy, often more severe than in late-onset cases [9-11]. In late-onset patients, the therapy shows some benefits (e.g., improvements in walking distance and respiratory function) [12,13], but skeletal muscle weakness often persists [14].

A better understanding of the underlying pathology may provide one key to improving therapy for skeletal muscle. The muscle biopsy, with all of its shortcomings, has become an irreplaceable tool for exactly this purpose. By analyzing biopsies, we have previously demonstrated that dysfunction of macroautophagy (often referred to as autophagy), a major intracellular lysosome-dependent degradation pathway [15,16], contributes significantly to the pathogenesis of the disease and interferes with delivery of the drug to the lysosomes [17-21]. The accumulation of autophagic debris - likely resulting from impaired fusion of lysosomes and autophagosomes (the vesicles which bring substrates and worn-out organelles to lysosomes) - is now a well-recognized phenomenon in Pompe disease as well as in other lysosomal storage disorders [22].

Here we present yet another pathological feature in the diseased muscle fibers: widespread accumulation of autofluorescent lipofuscin inclusions, which have previously been recognized as a sign of ageing [23,24]. Analysis of muscle biopsies from late-onset patients - including juveniles identified through new born screening (NBS) - demonstrated the pervasiveness of this pathology among all age groups.

## Methods

### Patient cohort

Muscle biopsies from 22 patients (11 male, 11 female) with late-onset Pompe disease (LOPD) and 1 (male) patient with atypical infantile-onset form were taken at Duke University Medical Center (Durham, NC, USA; 13 patients), the National Taiwan University Hospital (Taipei, Taiwan; 7 patients), and Rambam Medical Center (Haifa, Israel; 3 patients). Prior to biopsy, written informed consent was provided by the respective parents/guardians for all patients under institutional review board-approved protocols.

The cases from the Pompe program at Duke University included 12 patients with late-onset (adult) disease and an additional patient with atypical infantile form (D3; Tables 1 and 2). Patient D4 had been previously described after two years on therapy [25]. The Taiwanese cases included five patients identified through newborn screening (NBS) (NBSL2, NBSL6, NBSL9, NBSL15, and NBSL16) and two juvenile patients, one diagnosed clinically (CLINM) and the other (NBSL9a) through a family study for patient NBSL9 (Tables 2 and 3). Biopsies from patients NBSL2, NBSL6, NBSL9, NBSL9a, and NBSL15 were described previously [20,26]. We revisited these cases and performed additional confocal analyses of immunostained single fibers (see below). The Israeli cases included three juvenile-onset patients (HM1, HM3, and HM5; Table 2). All but one (D3) of the patients received alglucosidase alfa biweekly by infusion at 20 mg/kg (U.S. Prescribing Information, Genzyme Corp., 2006). The dosage was increased to 40 mg/kg in patient D3 due to frequent falls and regression in motor skills. In addition to histopathological and longitudinal clinical information, mutation data were collected for all patients (Additional file 1: Table S1).

**Table 1:** Adult-onset patients

**Patient ID**	**Onset, diagnosis (Age; y)**	**ERT start (Age; y)**	**Biopsy site; time on ERT (y)**	**Pathology/% fibers with lipofuscin inclusions**	**Clinical status (Current age)**
D4	27, 39	61	Forearm; 5-6	Mild lysosomal expansion in 2-4% of fibers; autophagic accumulation and inclusions in < 5% of fibers	Decreased strength; relies on wheelchair; can take a few steps (67 y)
D7	35, 61	62	VL^‡^; pre-treatment	Moderate lysosomal expansion in most fibers; autophagic accumulation and inclusions in ~33% of fibers	Relies on BIPAP at night; limited capacity for physical activity; independently ambulatory (62 y)
D8	35, 47	52	VL; 6*	Mild lysosomal expansion; autophagic accumulation and inclusions in ~42% of fibers	Relies on BiPAP; difficulty with stairs and getting out of the car and off the floor; relies on walker (58 y)
D9	10, 35	41	VL; 6-7	Mild-to-moderate lysosomal expansion in almost every fiber; autophagic accumulation in ~44% of fibers (20% with inclusions)	CPAP for sleep apnea; ambulatory with cane and walker (48 y)
D10	51 or 52, 54	54	VL; 5	Autophagic accumulation in ~19% of fibers; ~5% of fibers are destroyed**; inclusions are in <1% of fibers	Ambulatory with cane; difficulty with stairs and getting out of the car and off the floor (59 y)
D12^†^	48, 62	63	VL; 2	Mild lysosomal expansion; most fibers are normal; autophagic accumulation and inclusions are in < 5% of fibers	Uses BiPAP at night; ambulatory (65 y)
D13^†^	43, 43	46	VL; 2	Normal biopsy	Trunk weakness, lower back pain; ambulatory (48 y)
D14	51, 52	57	VL; 5	Moderate lysosomal expansion; autophagic accumulation and inclusions in ~30% of fibers	Uses walker periodically (62 y)
D15	22, 41	N/A	VL; 7	Mild-to-moderate lysosomal expansion; autophagic accumulation in ~46% of fibers (14% with inclusions)	Proximal weakness in upper and lower limbs; ambulatory with cane; relies on BiPAP; severe respiratory insufficiency (49 y)
D16	mid teens, 17	28	VL; 7	Mild lysosomal expansion; autophagic accumulation in ~16% of fibers; inclusions are in < 5% of fibers	Proximal weakness in upper and lower limbs; uses BiPAP at night; falls; difficulty climbing stairs (35 y)
D17	late 20s, 55	55	VL; 4	Autophagic accumulation with inclusions in ~20% of fibers	Proximal weakness in upper and lower limbs; ambulatory with cane or scooter; relies on BiPAP (59 y)
D19	39, 45	56	VL; 5	Autophagic accumulation with inclusions in ~25% of fibers	Weakness of the hip extensors and hip abductors; independently ambulatory; difficulty getting up from supine position (61 y)

*Increased dose to 30 mg/kg since 12/2011.

^†^D12 and D13 are siblings and D8 and D9 are siblings.

^‡^Vastus Lateralis.

**the fibers lacking recognizable myofibrillar structures are classified as “destroyed”.

**Table 2:** Atypical infantile-onset and juvenile-onset patients

**Patient ID**	**Onset, diagnosis (Age)**	**ERT start (Age)**	**Biopsy site; time on ERT**	**Pathology/% fibers with lipofuscin inclusions**	**Clinical status (Current age)**
D3*	5 mo	1 y, 4 mo	VL^†^; 3 y, 10 mo	Mild-to-moderate lysosomal expansion in most fibers; autophagic accumulation with inclusions in ~88% of fibers	Relies on powerchair; feeds orally; surgery for chronic right hip dislocation and left hip subluxation; no pulmonary compromise (5 y)
	1 y, 3 mo				
CLINM	13 y^¶^	13.6 y	Quad; 8 mo	Normal biopsy	Frequent low back pain; no difficulties in college gym classes (18.8 y)
	13.5 y				
HM1	4 mo^‡^	3 y	Quad; 6 y	Prominent lysosomal expansion in ~30% of fibers; atrophy; autophagic accumulation with Inclusions in ~77% of fibers; ~15% of fibers are completely destroyed	Wheelchair bound; respiratory failure; uses BiPAP at night; underwent several rounds of ITI due to high titer antibodies; progressive motor deterioration since 6 years of age (11 y)
	4 mo				
HM3	7 mo^‡^	10 y	Quad; 6 y	Most fibers completely destroyed; extensive damage obscures underlying pathology	Severe progressive lower limb muscle weakness; difficulty in walking and climbing stairs; non-compliance to ERT: stopped therapy for 6 months at age 14 y (18 y)
	7 mo				
HM5	5 y	6.5 y	Quad; 7 y	~ 80% of fibers completely destroyed, autophagic accumulation with inclusions in the remaining fibers	Motor deterioration; difficulty in walking and climbing stairs; uses BiPAP at night; respiratory failure; (14.5 y)
	~5 y				
NBSL9a^§^	6.5 y	7 y	Quad; baseline	Mild-to-moderate lysosomal expansion; inclusions in almost every fiber	Less endurance (10.7 y)
	6.5 y				

*Diagnosed with atypical infantile form of Pompe disease (no cardiac involvement).

^†^Vastus Lateralis.

^¶^Examined because of abnormal liver function test noted during routine check-up.

^‡^Examined because of family history.

^§^Older sibling of newborn screening patient NBSL9 (see Table 3); diagnosed during a family study.

**Table 3:** Patients identified through newborn screening

**Patient ID**	**Onset, diagnosis (Age)**	**ERT start (Age)**	**Biopsy site; time on ERT**	**Pathology/% fibers with lipofuscin inclusions Pretreatment**	**Pathology/% fibers with lipofuscin inclusions**	**Clinical status (Current age)**
					**Follow-up**	
NBSL2	36 mo, ~12 d	3 y	Quad; baseline	Mild lysosomal expansion; autophagic accumulation and inclusions in ~85% of fibers	NA	Less endurance (5.9 y)
NBSL6	34 mo, ~9 d	2.8 y	Quad; 6 mo	NA	Normal biopsy	In preschool, no difficulties (5.2 y)
NBSL9*	1.5 mo, 14 d	1.5 mo	Quad; baseline & 6 mo	Autophagic accumulation in 12.5% of fibers; inclusions are in < 1% fibers	Mild lysosomal expansion in ~15% of fibers	Can jump on one foot (4.7 y)
NBSL15	2.8 mo, ~3 d	2.8 mo	Quad; baseline & 6 mo	**Inclusions in ~10% of fibers	Mild-to-moderate lysosomal expansion in 10% of fibers; autophagic accumulation in ~15% of fibers; many normal fibers	Runs quickly, can jump using two feet (2.5 y)
NBSL16	4.5 mo, ~3 d	4.5 mo	Quad; baseline & 7 mo	Mild-to-moderate lysosomal expansion; autophagic accumulation with inclusions in ~10% of fibers	Normal biopsy	Runs quickly; can jump using two feet (2.1 y)

*Younger sibling of the juvenile-onset patient NBSL9a.

**this specimen was not suitable for immunostaining; autofluorescent inclusions were detected in unstained fibers.

### Tissue processing, staining, and microscopy

Biopsy samples were processed for routine histology and for immunostaining with the lysosomal marker LAMP2 and the autophagosomal marker LC3. Haematoxylin and eosin (H&E) and periodic-acid Schiff diastase (PAS-D) staining were performed according to standard procedures. LAMP2/LC3 immunostaining was performed on isolated muscle fibers as previously described [27]. The following primary antibodies were used: anti-LC3 (1:250; provided by Dr. Takashi Ueno, Juntendo University School of Medicine, Japan) and mouse anti-human LAMP2 monoclonal antibody (1:100; BD Biosciences Pharmingen, San Diego, CA). Alexa Fluor® 488 and 568 secondary antibodies were purchased from Invitrogen™ (Carlsbad, CA). For each patient, approximately 100 fibers were analysed by confocal microscopy (Zeiss LSM 510 META); the numbers of fibers with autophagic pathology, lysosomal abnormalities, and autofluorescent inclusions were counted. Additionally, lipid-staining techniques (Sudan Black B and Oil Red O; both from Sigma Aldrich, Saint Louis, MO) were used to analyse single fibers. Sudan Black B staining for lipofuscin was performed as described [28] with some modifications: to achieve sufficient staining, paraformaldehyde-fixed fibers [27] were stained for two hours (rather than 2 to 8 minutes as suggested in the original protocol) with 0.7% Sudan Black B dissolved in 70% ethanol. Two to three quick rinses in 50% and 70% ethanol were then performed, followed by several washes with PBS to remove excess stain. The fibers were analysed by confocal microscopy to verify the quenching of fluorescence. Oil Red O staining was performed as described [29].

### Microscopy characterization of lipofuscin and Fluorescence Lifetime Imaging (FLIM)

DIC contrast images and single autofluorescence images of LOPD inclusions were collected on a Leica SP5 NLO confocal system (Leica Microsystems) with excitation at 488 nm or the indicated wavelength. Second Harmonic Generation and 2-photon excited fluorescence were recorded on the same system with excitation at 870 nm provided by a 3 W MaiTai HP Ti:sapphire pulsed laser (Spectra-Physics, Santa Clara, CA) as described [30]. Fluorescence emission spectra were collected with 2-photon excitation at 840 nm. Emission was collected from 400 to 680 nm in 40 passages of 7 nm bandwidth and displayed with the Leica LAS AF software.

Time-resolved Fluorescence Lifetime imaging (FLIM) was done on the Leica SP5 NLO coupled to a PicoQuant SMD platform with Time-Tagged Time-resolved measurements. Two-photon excitation wavelength was 840 nm. Emitted photons were separated by a dichroic cube into two bandpasses, 470–550 nm and 607–683 nm, and were collected in two Avalanche Photo-Diodes detectors. Photon counting and picosecond timing was done in the PicoQuant PicoHarp300. Pre-FLIM images were collected on the SP5 and provided the image scale while all FLIM parameters were calculated in the PicoQuant SymphoTime software. Unstained fibers from muscle biopsy of patient NBSL9a and from *gastrocnemius* muscle of a 10.5-month-old GAA knockout mouse (GAA-KO; [31]) were analyzed. The samples were fixed with 4% p-formaldehyde and mounted in PBS. Average lifetimes (τ) were calculated for a region surrounding the inclusions by curve-fitting the data with 2 components: τ _avge_ = [A_1_τ_1_+ A_2_τ_2_]/A1 + A2. For each sample, four spectra and four FLIM images were recorded. Data were consistent from image to image.

Representative images from each of the three groups of patients were selected for analysis. Quantification of lipofuscin particles was performed with ImageJ (version 1.46r; Wayne Rasband, National Institutes of Health, Bethesda, MD; http://imagej.nih.gov/ij). The area occupied by the inclusions was expressed as a percentage of total autophagic area and as a percentage of total image area.

Animal care and experiments were conducted in accordance with the National Institutes of Health Guide for the Care and Use of Laboratory Animals.

## Results

We examined the clinical status and muscle pathology of 23 patients on ERT. With the exception of one patient with atypical infantile-onset disease, the remaining 22 individuals were classified as LOPD patients; however, these patients differed dramatically in clinical manifestations and age at diagnosis, ranging from infancy in patients identified through NBS to adolescence and late adulthood. Therefore, the data were analyzed separately for each group (Tables 1, 2, and 3).

**Figure 1:**
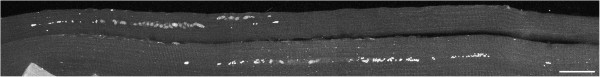
**Montage of confocal fluorescence images of unstained fibers from patient NBSL9a, showing numerous autofluorescent inclusions, single and in clusters, in the core of two fibers.** Bar: 50 μm.

The most striking finding in this study was the presence of large, irregularly shaped autofluorescent inclusions in muscle biopsies from 17 out of 23 patients (~74%). The inclusions were most conspicuous in 14 patients (~61%; Additional file 2: Table S2), and in many fibers they were the predominant pathology. In some patients (i.e., pts. D3, HM1, NBSL2, and NBSL9a) more than 75% of fibers contained these structures, which when adjacent, can extend (with or without interruption) up to several hundred microns along the length of the fiber (Figure 1).

### Characterization of autofluorescent inclusions in LOPD patients

**Figure 2:**
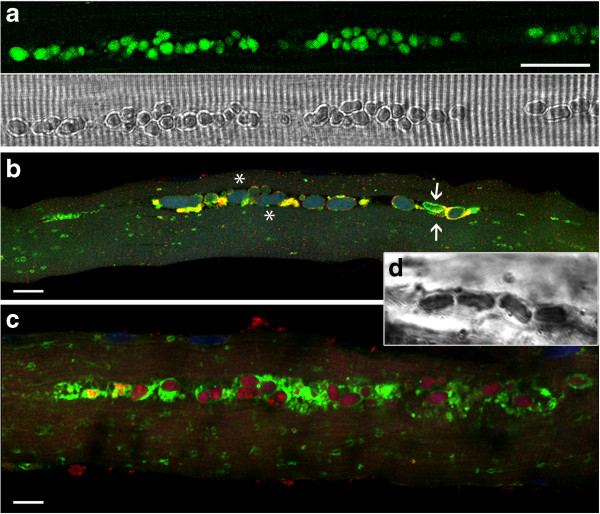
**Autofluorescent lipofusin inclusions in muscle biopsies from LOPD. (a)** LOPD fiber (pt. NBSL9a) viewed in fluorescence (top) and transmitted light (bottom) shows autofluorescent inclusions directly surrounded by myofibrils. Fluorescence was excited at 488 nm and collected from 467 to 499 nm. Transmitted light is with DIC contrast. Bar: 25 μm. **(b)** LOPD fiber (pt. NBSL2) with prominent inclusions. The fiber was stained with LAMP2 (lysosomes: green) and LC3 (autophagosomes: red). Some inclusions are seen within the lysosome or autolysosome (arrows) whereas others appear free in the cytoplasm (asterisks). Bar: 10 μm. **(c)** Autofluorescent inclusions stain positive for Oil Red. The fiber (isolated from muscle biopsy of pt. D3) was also stained with LAMP2 (green). Bar: 10 μm. **(d)** Sudan Black B staining demonstrates lipofuscin accumulation in a fiber from pt. D3. Bar: 10 μm.

Individually, the inclusions reached up to 8–10 μm in length and showed contrast in transmitted light microscopy (Figure 2a); these structures were often aligned throughout the core of the fiber (or occasionally clustered), and were commonly found within the area of autophagic buildup (Figure 2b). Indeed, the inclusions were frequently localized within LAMP-positive lysosomes or LAMP/LC3-double positive autolysosomes (vesicles formed by autophagosomal-lysosomal fusion); however, some fibers contained LAMP/LC3-negative inclusions, perhaps released into the cytoplasm due to lysosomal or autolysosomal rupture (Figure 2b asterisks). The autofluorescence and shape of these particles suggested that they consist of lipofuscin, an age-related, intralysosomal indigestible material found primarily in post-mitotic cells [32]. Further microscopy analysis was done to determine whether this is the case.

**Figure 3:**
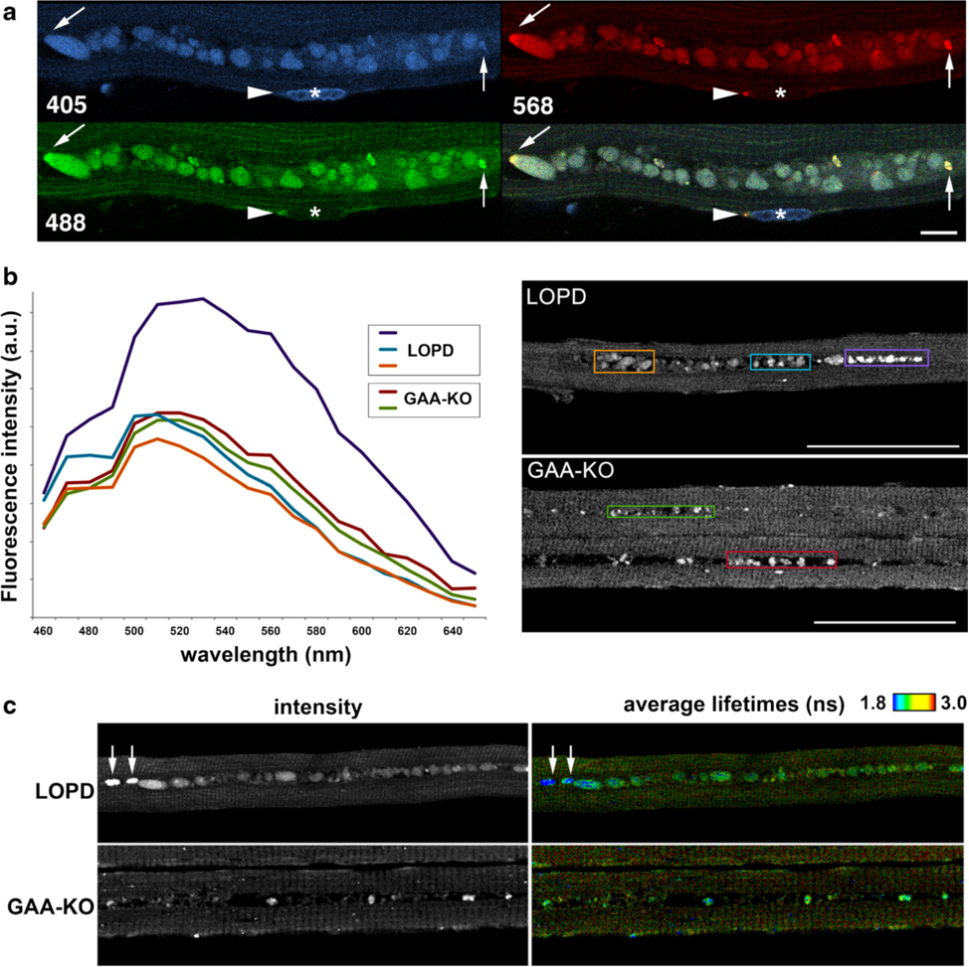
**Analysis of inclusions in muscle biopsies from an LOPD patient (NBSL9a) and a GAA-KO mouse. (a)** Confocal images of a muscle fiber from a LOPD biopsy with excitation at 405, 488, and 568 nm respectively. The last panel shows the sum of the three images. Autofluorescent particles are excited by each of the wavelengths while a Hoechst-stained nucleus (asterisk) is only excited at 405 nm. An arrowhead points to a small normal-looking lysosome at a pole of the nucleus while an arrow points to the end of the particle row with a small brighter area. **(b)** Two-photon excited fluorescence of LOPD and GAA-KO fibers recorded in spectral mode on a confocal microscope. Fluorescence emission spectra from 460 to 660 nm were displayed for the areas within colored boxes and plotted in Excel. There are minor differences between the human and mouse samples - LOPD fibers have particles that stand out in brightness and are slightly red-shifted (purple box and spectrum); these particles are commonly found at the end of the row of inclusions (see also arrows in panel **a)**. Background autofluorescence corresponds to mitochondria in I bands [30]. **(c)** FLIM analysis confirms the heterogeneity of autofluorescent particles in both GAA-KO and LOPD fibers. Left panels show the intensity of fluorescence emission while right panels are pseudo-colored to represent average lifetimes. The bright particles in the LOPD fiber (arrows) are similar to those in the purple box shown in **b**; their average lifetime is shorter (blue color). The wide spectra **(a & b)** support the notion that the inclusions consist of lipofuscin; FLIM analysis suggests that the particles may mature as the disease progresses. Bars: 10 μm **(a)**; 50 μm **(b)**.

The inclusions stained positive for Sudan Black B and Oil Red O - both accepted lipid markers - and their autofluorescence was quenched by Sudan Black B (Figure 2c and d), arguably the most specific test for lipofuscin identification [28]. The inclusions displayed a wide-spectrum autofluorescence: they were excited by light over the visible spectrum from 405 nm to 568 nm (Figure 3a). Furthermore, when excited at 840 nm in two-photon fluorescence, the inclusions had a wide emission spectrum similar to that of human retina lipofuscin [33] with a shoulder at 470 nm and maxima around 510–520 nm. A similar autofluorescence spectrum was obtained for autofluorescent particles in the autophagic areas of GAA-KO mouse muscles (Figure 3b). These broad spectral properties are, again, consistent with those of lipofuscin granules [23,24,34]. Taken together, the data indicated that the inclusions in patients’ biopsies represent lipofuscin – a biomarker of aging in human skeletal muscle [35].

To obtain more information about the homogeneity of the particles, we used fluorescence lifetime imaging (FLIM) which can distinguish different molecules fluorescing at the same wavelength [33]. Experimental data were well fitted with a 2-component model, a short lifetime component (0.7 nsec for both LOPD and GAA-KO fibers) and a longer lifetime component (2.9 nsecs for LOPD and 3.2 nsecs for GAA-KO). FLIM representation showed that there is some heterogeneity within the same sample. Particles found at the ends of the inclusion rows in LOPD (arrows in Figure 3c) had a higher contribution of the short lifetime component, as indicated by the different color; these end particles were also brighter. There were also differences between human and mouse samples (Figure 3c): average lifetimes were 1.8 nsec for the inclusions in GAA-KO fibers and 1.4 nsec for those in LOPD fibers. Thus, FLIM reveals heterogeneity of the particles, suggesting that the lipofuscin inclusions (or their environment) may evolve as the disease progresses and that the composition of lipofuscin may be species-dependent.

**Figure 4:**
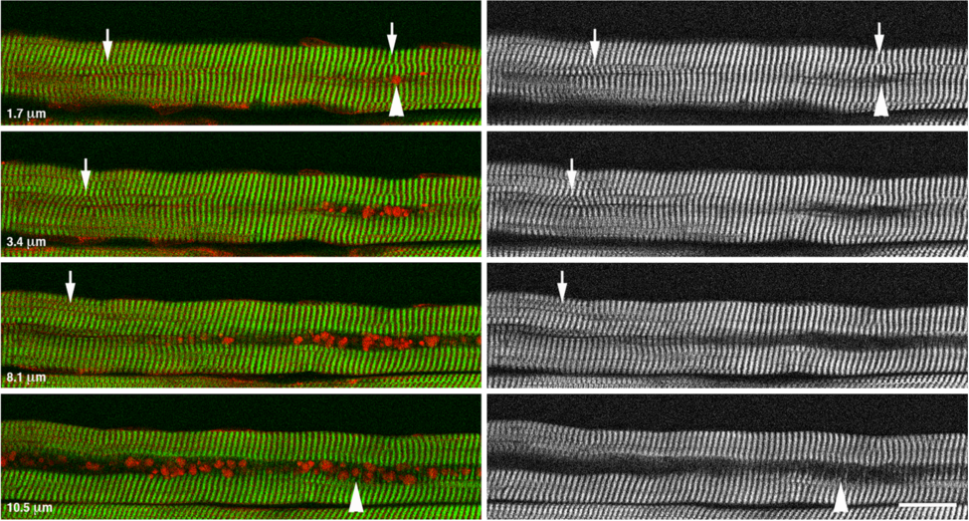
**Second Harmonic Generation (SHG) microscopy of a single fiber from an LOPD muscle biopsy.** Stacks of SHG and autofluorescence images were recorded with a step of 0.85 μm to image the whole fiber from top to bottom. SHG (green) shows myosin bands, whereas autofluorescence (red) shows the inclusions. Several images from one series are shown here. The distance from the top of the fiber is indicated. As the focal plane encounters the first inclusions, the interruption of the myosin bands is clear. The interruption becomes the “black hole” of autophagic areas (see text). As the focal plane reaches the other side of the particles, thin partial myosin bands are seen. Arrowheads point to the interruptions, total or partial of the myosin bands; arrows highlight defects in the myofibril alignment. Bar: 25 μm.

We have also used Second Harmonic Generation (SHG) microscopy [36] - a technique which allows visualization of myosin bands in unstained muscle samples [37] - to see the effect of the inclusions on the overall organization of muscle fibers. The inclusions were frequently found within a “black hole” (Figure 4 and Additional file 3: Figure S1) similar to that seen in aged GAA-KO muscle fibers; in the GAA-KO the autophagic buildup is located within these holes [30]. SHG imaging showed that the inclusions disrupt the muscle fiber architecture by interrupting the contractile myofibrils (Figure 4, arrowheads). Furthermore, myofibrillar defects are also obvious in the adjacent areas (Figure 4, arrows).

### Adult-onset patients

Twelve out of 23 cases were adult-onset patients (D4, D7, D8, D9, D10, D12, D13, D14, D15, D16, D17, and D19). Of these twelve, nine patients share a splice site mutation (c.-32-13 T > G), commonly found in late-onset patients [38,39]. Mutation data were not available for D15, D16, and D19 (Additional file 1: Table S1). A single biopsy, taken 2–7 years after ERT initiation (median: 6 years; see Table 1), was available for eleven of these patients; a pre-treatment biopsy was obtained from the remaining patient (D15), who had just started therapy at the time of the study. The median age at biopsy was 59 years (range: 35–66) (Table 1, distributions shown in Additional file 4: Figure S2).

Three patients - D4, D12, and D13 (currently aged 66, 65, and 48, respectively) - showed minimal or no pathology by both immunostaining of single muscle fibers and routine histological examination of muscle sections (Table 1). After 2 years on therapy, both D12 and D13 remained ambulatory and experienced only mild symptoms (i.e., lower back pain and/or muscle weakness), indicating that there is a good correlation between their clinical status and the condition of their muscle tissue. In contrast, pt. D4 appears to be more affected than her left forearm biopsy would indicate (i.e., she relies on a wheelchair for daily activity), suggesting that the site of biopsy was inadequate. Of note, this patient was described previously after 2 years of therapy [25]; at that time, significant gains in motor and pulmonary function were reported, and her condition has since stabilized.

**Figure 5:**
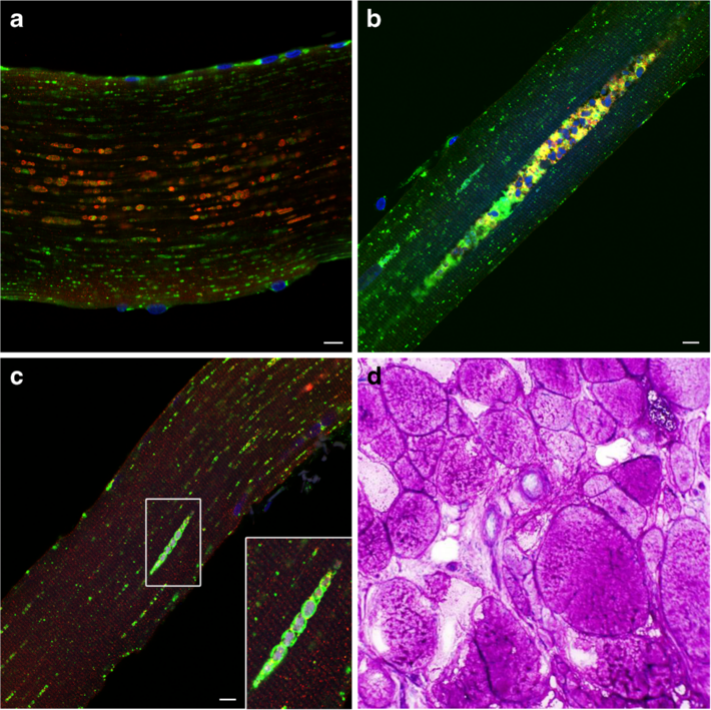
**Autophagic abnormalities and autofluorescent inclusions in adult-onset patients. a-c:** Muscle fibers were stained for lysosomal marker LAMP2 (green) and autophagosomal marker LC3 (red). **(a)** LOPD fiber (pt. D16) shows mild lysosomal enlargement and clusters of autophagosomes. **(b)** Autophagic buildup and autofluorescent inclusions represent a major pathology in this fiber (pt. D7). **(c)** Autofluorescent inclusions are seen within the LAMP2-positive structures (pt. D7). Bar in **a-c**: 10 μm. **(d)** Both autophagic buildup and inclusions are missed by routine histology; the image shows epon-embedded PAS-stained section of muscle biopsy from pt. D15 (10x).

Analysis of single muscle fibers of the remaining nine patients demonstrated three features - lysosomal enlargement, autophagic abnormalities, and autofluorescent inclusions - all to different degrees (D7, D8, D9, D10, D14, D15, D16, D17, and D19). When present, lysosomal pathology (defined here as lysosomal expansion in the area outside autophagic buildup or in the fibers free from buildup) was mild to moderate (lysosomal diameter less than 2 μm or between 2 and 5 μm, respectively) and did not appear to disrupt muscle architecture. In contrast, autophagic abnormalities - ranging from clusters of enlarged autophagosomes to larger buildup areas - were much more pervasive (i.e., present in 16% to 46% of fibers) and often constituted the only pathology within individual fibers (Figure 5a and b; shown for D7 and D16). Autophagic abnormalities were easily detectable by single fiber analysis, but were missed by routine histology in five cases (shown for pt. D15 in Figure 5d). Autofluorescent inclusions either within clearly defined LAMP-positive structures (Figure 5c) or within less recognizable entities in large autophagic areas were prominent in seven of the nine patients and occupied up to 38% of the autophagic buildup area (D7, D8, D9, D14, D15, D17, D19; Table 1 and Additional file 2: Table S2). The inclusions remained unnoticed by routine histology. All patients in this group of nine experience walking difficulties, and many rely on BiPAP or CPAP for respiratory assistance. Considering the unremarkable lysosomal pathology, the non-contractile lipofuscin inclusions and autophagic buildup are likely to contribute to the patients’ clinical manifestations.

**Figure 6:**
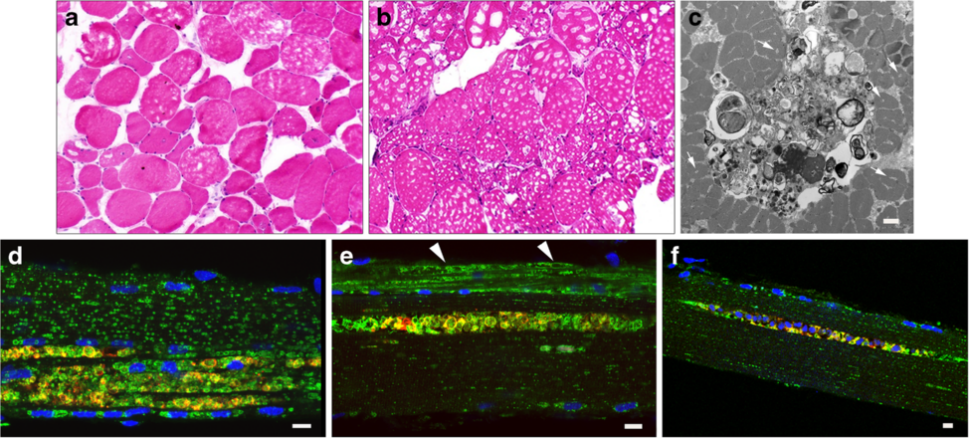
**Analysis of muscle biopsies from an adult-onset patient D9. (a)** H&E stained section of the first biopsy (taken 6 years prior to initiation of ERT) shows vacuolation in ~20-25% fibers (10x). The second biopsy was performed after 6 years of ERT **(b-f). (b)** H&E staining shows mostly vacuolated fibers (10x) (note, some of the large “holes” are likely freeze artefacts). **(c)** EM demonstrates the presence of autophagic buildup and “pale” areas (arrows) in the surrounding relatively well preserved fibers (transverse section). Bar: 2 μm. **(d-f)** Muscle fibers were stained for lysosomal marker LAMP2 (green) and autophagosomal marker LC3 (red). Nuclei are stained with Hoechst (blue). LAMP2/LC3 staining demonstrates prominent autophagic accumulation with inclusions in most fibers; these abnormalities are commonly seen in fibers with mild **(d)** or no **(e** and **f)** lysosomal enlargement. Prominent lysosomal enlargement is seen in occasional fibers (**e**; arrowheads). Bar: 10 μm.

Interestingly, one case (D9) may provide insight into the progression of pathology during the course of ERT. The patient had biopsies taken before (age 35) and following 6 years of ERT; the findings from these two biopsies, which were analyzed by routine histology and EM, differ dramatically. The tissue from the first biopsy (quadriceps; Figure 6a) was less affected (i.e., it showed fewer vacuolated fibers; compare Figure 6a and b), but glycogen in membrane-bound vacuoles (lysosomes) was readily identifiable by EM (not shown). In the second biopsy, intralysosomal glycogen was difficult to identify; instead, pools of pale material and autophagic buildup were detected in many fibers (Figure 6c). Single fiber analysis from this second biopsy confirmed the presence of inclusions and autophagic accumulation in ~ 44% fibers (Table 1 and Figure 6d-f); only occasional fibers still showed significant lysosomal enlargement (Figure 6e; arrowheads). It appears that ERT resolved lysosomal pathology in the majority of fibers, whereas autophagic accumulation and inclusions - the major secondary abnormalities - persisted.

### Patients identified by newborn screening

Since autofluorescent inclusions, often associated with autophagic buildup, were a prominent feature in most adult-onset patients, we wondered how early they developed in LOPD patients. To address this question, we took advantage of a rare opportunity afforded by the newborn screening (NBS) program in Taiwan [40,41], which allows early diagnosis and treatment initiation following the first signs of the disease. We have analyzed single fibers from the biopsies of patients NBSL6, NBSL15, and NBSL16 and revisited two additional patients (NBSL2, NBSL9) whom we had previously described (Table 3) [20]. These patients started therapy between 1.5 months and 3 years of age. Pretreatment biopsies (available for all except for NBSL6) again showed a different combination of lysosomal and autophagic defects, and lipofuscin inclusions (Table 3). The inclusions occupied 36-58% of the autophagic areas and were present in ~85% of NBSL2 fibers, in ~10% of NBSL15 and NBSL16 fibers, and were rare (<1% fibers) in NBSL9 (Figure 2b; Table 3 and Additional file 2: Table S2).

For four of the five patients (NBSL6, NBSL9, NBSL15, and NBSL16), biopsies taken after 6 months of ERT were available for single fiber analysis. Consistent with previous data [20,26], most of these patients responded remarkably well to therapy and exhibited normal or near normal fiber morphology (for example, NBSL6, NBSL9, NBSL16). Interestingly, the patient whose pre-treatment biopsy (the only one available for analysis) was most affected by inclusions - NBSL2 – appeared to have less dramatic clinical improvement (Table 3).

Most of these pre- and post-treatment biopsies were previously analyzed by EM and histological analysis, which was performed by high-resolution light microscopy [42]. Autophagic debris was easily detectable and reported [26]; the inclusions, however, were overlooked, but are clearly visible in retrospect. Of note, the follow-up biopsies in this group of patients were taken after only 6–7 months on ERT. Long-term follow-up biopsies may help establish the correlation between the clinical status and the extent of inclusions.

### Juvenile-onset patients

In a group of juvenile-onset patients - three Israeli patients (HM1, HM3, and HM5) and two Taiwanese patients (CLINM and NBSL9a; Table 2) - there was remarkable heterogeneity in both the pathology and clinical status. Two of the Israeli patients, HM3 and HM5, exhibited initial clinical improvement followed by rapid deterioration after 6 to 7 years on therapy. HM1, who developed antibodies to the replacement enzyme and required immune-tolerance induction (ITI) therapy [43], experienced motor decline after three years on ERT. In contrast, the Taiwanese patients do not show such deterioration. For example, patient CLINM - who started therapy the latest - shows no clinical signs of the disease except for frequent lower back pain after five years on ERT.

**Figure 7:**
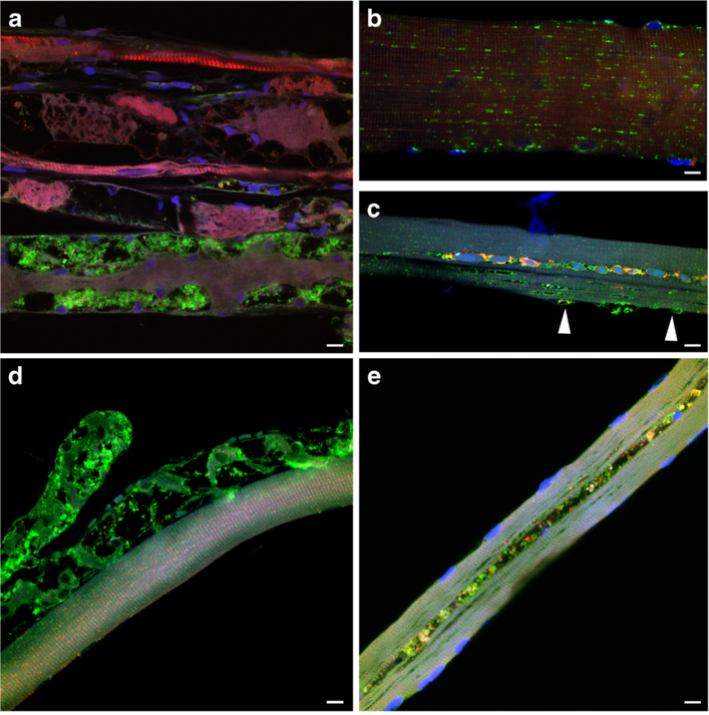
**Analysis of muscle biopsies from juvenile-onset patients.** Muscle fibers were stained for lysosomal marker LAMP2 (green) and autophagosomal marker LC3 (red). LAMP2/LC3 immunostaining demonstrates variability of muscle fiber involvement: completely destroyed fibers in pts. HM3 **(a)** and HM1 **(d)**; a typical well- preserved fiber in pt. CLINM **(b)**; fibers with inclusions in pts. HM1 and HM5 **(c** and **e** respectively), and a fiber with lysosomal enlargement in pt. HM1 **(c**; arrowheads). Bar: 10 μm.

In most cases, the pathology adequately reflects the spectrum of clinical outcomes observed in this group; whereas most fibers were destroyed in the biopsy from HM3 (Figure 7a), CLINM exhibited a completely normal biopsy following 8 months of therapy at 14.4 years of age (Figure 7b). Apart from these two extreme cases, the remaining Israeli patients (HM1 and HM5) showed a wide range of pathology: completely preserved fibers alongside fibers with prominent lysosomal enlargement (diameters exceeding 7 μm; Figure 7c; arrowheads; pt. HM1), autophagic buildup, and autofluorescent inclusions (Figure 7c-e; Table 2 and Additional file 5: Figure S3).

Lipofuscin inclusions were particularly striking in NBSL9a; they were seen in almost every fiber and often found in otherwise normal looking fibers (Figures 1, 2a, and 3; Additional file 2: Table S2). Again, in retrospect, the inclusions are easily recognizable by high-resolution light microscopy [26]. Clinically, this patient shows decreased endurance. Importantly, the younger sibling of NBSL9a, who was diagnosed through the NBS program and began therapy at a much younger age (1.5 months instead of 7 years of age; Tables 3 and 2), does not show any symptoms of the disease.

**Figure 8:**
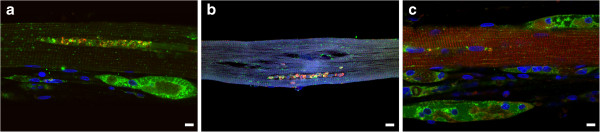
**Analysis of muscle biopsy from a patient (D3) with atypical infantile-onset Pompe disease.** Muscle fibers were stained for lysosomal marker LAMP2 (green) and autophagosomal marker LC3 (red). LAMP2/LC3 immunostaining demonstrates variability of muscle fiber involvement: fibers with autophagic accumulation (for example, top fiber in panel **a)** and inclusions **(b)**, in addition to largely intact muscle fibers **(c)** interspersed with completely destroyed fibers **(a** and **c)**. Bar: 10 μm.

Finally, a biopsy from a five year-old patient with atypical infantile-onset Pompe disease (D3; Table 2) also showed prominent inclusions. In a sample taken after 4 years of therapy, inclusions were present in approximately 88% of fibers. As in many other cases, these structures were found either within areas of autophagic buildup or in isolated LC3- and LAMP2-positive structures (Figure 8a and b); relatively well preserved fibers can be seen next to completely destroyed ones (Figure 8a and c). Although the patient feeds orally and does not require respiratory support, he has persistent skeletal myopathy with scapular winging, scoliosis, and limb girdle weakness.

## Discussion

The utility of muscle biopsies in LOPD is rightly questioned in the Pompe disease community [44]. From the perspective of a clinician, muscle biopsies are not reliable for diagnostic purposes, do not always serve as a prognostic tool, and expose patients (particularly younger ones) to further discomfort and anesthesia risk. It is well known that different muscle groups and even fibers within the same muscle group exhibit high variability in the extent and severity of pathology, especially in late-onset cases. This heterogeneity makes it difficult to generalize findings from a single biopsy site and to establish an overall correlation between pathology and clinical status in a patient cohort. An example of the former is the normal biopsy we observed in patient D4, an elderly woman who remains symptomatic (as mentioned above, this mismatch is most likely due to sampling at a “wrong” site). As for the latter, the only association that can be made is that severe pathology invariably manifests in poor clinical status. We have recently shown this association in a group of infantile patients and now extend our findings to late-onset patients [11].

Considering the shortcomings of the muscle biopsy, there is a growing tendency to avoid this procedure. However, the muscle biopsy remains invaluable in at least one regard - understanding the pathogenesis of Pompe disease and the mechanisms of skeletal muscle damage; such information may justify the need for earlier therapy and assist in the development of a better therapy. Dysfunction of autophagy, first found in an animal model [17,31,45,46], was only recognized as a major secondary pathology after extensive analysis of single fibers from human biopsies [19,20,47]. In the current study, muscle biopsies from a large group of patients at different ages and stages of the disease enabled us to establish the presence of lipofuscin inclusions as yet another abnormality.

Abnormal inclusions in Pompe skeletal muscle have been reported as early as 1992 and in subsequent studies [20,48-51]. In retrospect, these structures - called “reducing body-like inclusions,” “lipofuscin debris”, “peculiar globular inclusions,” “acid phosphatase-positive globular inclusions” and our own “autofluorescent balloon-like structures”- are likely one and the same. The identification of this pathology in human biopsies has now allowed us to recognize similar (albeit much smaller) inclusions in our aging GAA knockout mice. It was suggested that the acid phosphatase-positive inclusions, which appeared as electron-dense globules by electron microscopy [50] may serve as a diagnostic marker for LOPD in cases when “typical vacuolated fibers are absent” in muscle biopsies [51].

In all cases, the structure and staining characteristics of these inclusions appear to be consistent with those of lipofuscin, an intralysosomal indigestible autofluorescent material. Confocal microscopy of isolated muscle fibers or muscle bundles is ideally suited for detection of these structures, but high-resolution light microscopy (HRLM) [42] is a close second. HRLM technique, which allows for excellent preservation of glycogen, can be easily adopted as a routine procedure to analyze muscle biopsies in Pompe disease. In fact, in retrospect, large lipofuscin deposits are clearly seen in some of our patients’ samples, which were processed by HRLM [26]; this outcome underscores the old adage - you see what you’re looking for.

Found primarily in terminally differentiated cells (i.e., neurons, cardiac myocytes, retinal pigment epithelium, and muscle cells), lipofuscin is a polymeric substance composed of oxidized and cross-linked proteins and lipid clusters, as well as carbohydrates and metals (especially redox-active iron) [32]. Accumulation of lipofuscin granules within postmitotic cells is a marker of cellular oxidative damage and aging [23,24,34,52,53]. According to the mitochondrial-lysosomal axis theory of ageing, mitochondrial stress and oxidative damage to cytosolic proteins lead to the formation of “biological garbage” - cross-linked proteins and lipids resistant to enzymatic degradation - which are then delivered to lysosomes through the autophagic pathway [53,54]. Aside from age-related lipofuscinogenesis, the pathological accumulation of lipofuscin has been implicated in amyotrophic lateral sclerosis [55], lysosomal storage diseases (e.g., neuronal ceroid lipofuscinosis, or Batten’s disease [56]), malnutrition, and muscular dystrophies [32,57,58]. Excessive lipofuscin accumulation in muscle has been reported in patients with chronic obstructive pulmonary disease [59] and in dystrophin-deficient DMD patients and mdx mice [60].

In Pompe disease, the accelerated production of lipofuscin is not a feature of advanced age. These deposits are prominent in the youngest LOPD patients in this study (e.g., NBSL15; 2.8 months of age) as well as in infantile patients on therapy, as we have previously reported [11]. It appears that the disease develops into a “muscle lipofuscinosis,” particularly in fibers free from lysosomal glycogen accumulation. The presence of lipofuscin in the diseased skeletal muscle is not completely surprising given the failure of the degradative system. We have previously shown in the GAA-KO mouse model that lysosomal glycogen storage leads to dysfunctional autophagy, accumulation of autophagic substrates, and impaired fusion between autophagic and lysosomal vesicles, thereby initiating the process of autophagic buildup which spreads throughout the fiber [21,45,61]. Given the unique role of autophagy in mitochondrial degradation [62], this autophagic dysfunction may result in the accumulation of worn-out mitochondria, which in turn would generate reactive oxygen species and perpetuate the production of lipofuscin [47].

Once formed, lipofuscin can reduce lysosomal degradative capacity and decrease the autophagic turn-over of damaged mitochondria, contributing to the vicious cycle of lipofuscinogenesis [32]. Perhaps this would account for the striking size and extent of inclusions seen in LOPD patient biopsies. In addition, it has been suggested that newly synthesized lysosomal enzymes are diverted to and squandered in lipofuscin-burdened lysosomes [32]. In Pompe disease, such a “sink” may affect the trafficking of the recombinant enzyme, similarly to what we described for entire areas of autophagic buildup [17,21]. Furthermore, lipofuscin - by its very definition - is not treatable by ERT. Prevention of excessive lipofuscin deposits or exocytosis of lipofuscin-laden lysosomes may be the only strategies to address this extensive and previously underappreciated pathology.

## Conclusions

The limitations of ERT underscore the need for a better understanding of the pathogenesis of skeletal muscle damage in PD, which has been viewed for years as simple enlargement of glycogen-filled lysosomes and lysosomal rupture. Just as the muscle biopsy has previously enabled us to uncover autophagic defects, the technique has now facilitated the identification of a related pathological feature, large lipofuscin inclusions often found within the area of autophagic accumulation. Furthermore, since these inclusions represent the predominant pathology within many fibers, the disease may in fact be characterized as a “muscle lipofuscinosis” and require a new approach to therapy. Muscle biopsies remain an invaluable material for the further analysis of the molecular composition of lipofuscin inclusions and their fate in ERT-treated patients. In fact, muscle biopsies are carried out for variety of reasons, and it would be prudent to utilize them to address the question.

## Abbreviations

ERT: Enzyme replacement therapy; Pompe disease; LOPD: Late-onset Pompe disease; GAA: Acid alpha-glucosidase; LAMP2: Lysosomal-associated membrane protein-2; LC3: Microtubule-associated protein light chain 3; H&E: Haematoxylin and eosin; PAS-D: Periodic-acid Schiff stain after diastase digestion; NBS: Newborn screening; ITI: Immune tolerance induction; FLIM: Fluorescence lifetime imaging; SHG: Second harmonic generation microscopy.

## Competing interests

The authors declare that they have no competing interests. Priya S. Kishnani has received research/grant support and honoraria from Genzyme Corporation and is a member of the Pompe and Gaucher Disease Registry Advisory Board for Genzyme Corporation. Yin-Hsiu Chien and Wuh-Liang Hwu have received research/grant support and honoraria from Genzyme Corporation. Benedikt Schoser has received honoraria from Genzyme Corporation and is a member of the Pompe Global Advisory Board.

## Authors’ contributions

EJF coordinated data collection, analyzed and interpreted data, and helped draft the manuscript; PSK, SA, and SP provided clinical information and muscle biopsy specimens from patients treated at Duke University; YHC and WLH provided clinical information and muscle biopsy specimens from patients treated in Taiwan; HM provided clinical information and muscle biopsy specimens from patients treated in Israel; ER performed spectral analysis and fluorescence lifetime imaging, and interpreted the data; BS and PSK analyzed and interpreted the data with NR; NR designed the study, generated data on autophagy and lipofuscin, analyzed and interpreted the data, and wrote the paper. All authors read and approved the final manuscript.

## Supplementary Material

Additional file 1: Table S1 Patients’ ethnicity and mutation data. Click here for file

Additional file 2: Table S2 Inclusions in LOPD patients. Click here for file

Additional file 3: Figure S1 Second Harmonic Generation (SHG) microscopy of a muscle biopsy from an LOPD patient. Unstained LOPD muscle fibers were excited at 870 nm in 2-photon mode. SHG images (which reveal myosin bands, in green) and 2P-excited fluorescence images (which reveal autofluorescent particles, in red) were recorded simultaneously. In panels a & b myosin bands are weaker but appear uninterrupted around the particles. In panels c & d “black holes” characteristic of areas of autophagic debris are very clear (see [30]). Stacks of SHG and autofluorescence images of the fiber in panel d are shown in Fig. 4 of the main text. Bar: 20 μm. Click here for file

Additional file 4: Figure S2 Time course of onset, diagnosis, and ERT initiation in adult-onset patients. Click here for file

Additional file 5: Figure S3 Phase contrast image of muscle fibers from a muscle biopsy of patient HM1. The image, which shows large lipofuscin deposits in one of the two fibers, was taken by wide field microscopy. Click here for file
