# Supplementary material for: The value of muscle biopsies in Pompe disease: identifying lipofuscin inclusions in juvenile- and adult-onset patients
Source: Acta Neuropathol Commun. 2014 Jan 2;2:2. doi: 10.1186/2051-5960-2-2 (PMC3892035; doi:10.1186/2051-5960-2-2)
Supplement: Additional file 1: Table S1 — Patients’ ethnicity and mutation data. [file 2051-5960-2-2-S1.doc]

**Supplementary Table 1. Patients’ ethnicity and mutation data**

| **Pt.ID/Sex**  **Ethnicity** | **Mutations** | **Comments*** |
| --- | --- | --- |
| D3/M  African Am. | 1: c.1447G>A (p.Gly483Arg) 2: c.-32-17_-32-10 deletion insertion **^†^** | 1: less severe  2: novel |
| D4/F  Caucasian | 1: c.-32-13T>G **^‡^**  2: c.1642G>T (p.Val548Phe); c.1880C>T (p.Ser627Phe) | 1: leaky splice mutation; mild;  2: severity unknown |
| D7/F  Caucasian | 1: c.-32-13T>G  2: c.2481+102_2646+31del (p.Gly828_Asn882del) | 1: leaky splice mutation; mild  2: very severe |
| D8/M  Caucasian | 1: c.-32-13T>G  2: c.1445C>G (p.Pro482Arg) | 1: leaky splice mutation; mild  2: potentially less severe |
| D9/F  Caucasian | 1: c.-32-13T>G  2: c.1445C>G (p.Pro482Arg) | 1: leaky splice mutation; mild  2: potentially less severe |
| D10/F  Caucasian | 1: c.-32-13T>G  2: c.2481+102_2646+31del (p.Gly828_Asn882del) | 1: leaky splice mutation; mild  2: very severe |
| D12/M  Caucasian | 1: c.-32-13T>G  2: c.1143delC | 1: leaky splice mutation; mild  2: novel |
| D13/M  Caucasian | 1: c.-32-13T>G (also has silent mutation: c.876C>T)  2: c.1143delC | 1: leaky splice mutation; mild 2: novel |
| D14/M  Caucasian | 1: c.-32-13T>G  2: c.1447G>A (p.Gly483Arg) | 1: leaky splice mutation; mild  2: less severe |
| D15/M  Caucasian | N/A | N/A |
| D16/F  Caucasian | N/A | N/A |
| D17/M  Caucasian | 1: c.-32-13T>G  2: c.2647-20T>G | 1: leaky splice mutation; mild 2: novel |
| D19/F  Caucasian | N/A | N/A |
| CLNM/M  Taiwanese | 1: c.1935C>A (p.Asp645Glu)  2: c.2238G>C (p.Trp746Cys) | 1: potentially less severe 2: potentially mild |
| NBSL2/M  Taiwanese | 1: c.2238G>C (p.Trp746Cys)  2: c.2662G>T (p.Glu888X) | 1: potentially mild  2: very severe |
| NBSL6/M  Taiwanese | 1: c.1935C>A (p.Asp645Glu)  2: c.752C>T, c.761C>T (p.Ser251Leu, p.Ser254Leu) | 1: potentially less severe  2: both non-pathogenic? |
| NBSL9/M  Taiwanese | 1: c.1935C>A (p.Asp645Glu)  2: c.2238G>C (p.Trp746Cys) | 1: potentially less severe 2: potentially mild |
| NBSL9a/F  Taiwanese | 1: c.1935C>A (p.Asp645Glu)  2: c.2238G>C (p.Trp746Cys) | 1: potentially less severe 2: potentially mild |
| NBSL15/M  Taiwanese | 1: c.872T>C (p.Leu291Pro)  2: c.1798C>T (p.Arg600Cys) | 1: potentially less severe 2: less severe |
| NBSL16/F  Taiwanese | 1: c.1634C>T (p.Pro545Leu)  2: c.1935C>A (p.Asp645Glu) | 1: less severe  2: potentially less severe |
| HM1/F  Israeli Druze | c.1064T>C (p.Leu355Pro); homozygous | potentially less severe |
| HM3/F  Arab Muslem | c.670C>T (p.Arg224Trp); homozygous | potentially less severe |
| HM5/F  Isra Israeli Druze | 1: c.1064T>C (p.Leu355Pro)  2: c.1210G>A (p.Asp404Asn) | 1: potentially less severe  2: potentially less severe |

* the effect of mutations on GAA function is presented as described on the Erasmus MC Rotterdam Pompe Center website ([www.pompecenter.nl](http://www.pompecenter.nl/))

**^†^** the novel deletion/insertion TCCCTGCTGAGCCTCCTACAGGCCTCCCGC overlaps with c.-32-13T>G; likely affects exon 2 splicing

**^‡^** the mutation found in over half of all adult Caucasian patients
