# Supplementary material for: The value of muscle biopsies in Pompe disease: identifying lipofuscin inclusions in juvenile- and adult-onset patients
Source: Acta Neuropathol Commun. 2014 Jan 2;2:2. doi: 10.1186/2051-5960-2-2 (PMC3892035; doi:10.1186/2051-5960-2-2)
Supplement: Additional file 2: Table S2 — Inclusions in LOPD patients. [file 2051-5960-2-2-S2.doc]

**Supplementary Table 2. Inclusions in LOPD patients**

| Patient ID | % area occupied by inclusions/total image area | % area occupied by inclusions/autophagic area | % area occupied by autophagic area/total image area | * % fibers with inclusions |
| --- | --- | --- | --- | --- |
| Adult-onset patients | | | | |
| D7 | 5.8 | 38.4 | 14.5 | 33 |
| D8 | 2.4 | 25.8 | 10.9 | 42 |
| D9 | 2.7 | 27.4 | 12.1 | 20 |
| D14 | 4.2 | 31.6 | 13.8 | 30 |
| D15 | 2.0 | 27.4 | 7.3 | 14 |
| D17 | 3.2 | 30.0 | 11.2 | 20 |
| D19 | 1.8 | 26.7 | 8.9 | 25 |
| Patients identified through Newborn Screening | | | |  |
| NBSL2  pre-treatment | 5.4 | 58.1 | 9.7 | 85 |
| NBSL15  pre-treatment | 4.3 | 51.2 | 8.2 | 10 |
| NBSL15  6 mo on ERT | 2.5 | 49.8 | 4.8 | 10 |
| NBSL16  pre-treatment | 1.5 | 35.9 | 4.0 | 10 |
| Atypical infantile-onset and juvenile-onset patients | | | |  |
| D3 | 7.2 | 44.7 | 16.8 | 88 |
| HM1 | 9.4 | 46.8 | 20.6 | 77 |
| HM5 | 12.2 | 44.8 | 23.3 | 20** |
| NBSL9a | 5.7 | 37.4 | 16.5 | 100 |

* the numbers in this column are also included in Tables 1, 2, and 3 of the main text.

** a subset of partially preserved fibers
