# Supplementary figures and images for: The value of muscle biopsies in Pompe disease: identifying lipofuscin inclusions in juvenile- and adult-onset patients
Source: Acta Neuropathol Commun. 2014 Jan 2;2:2. doi: 10.1186/2051-5960-2-2 (PMC3892035; doi:10.1186/2051-5960-2-2)

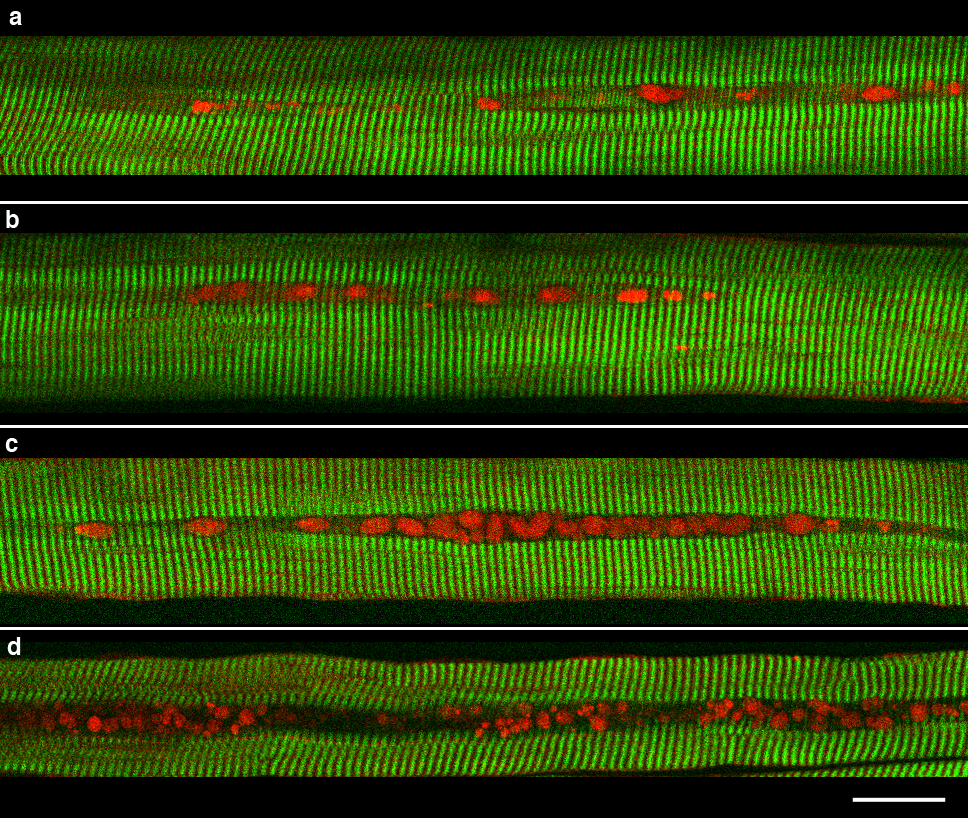

Supplement: Additional file 3: Figure S1 — Second Harmonic Generation (SHG) microscopy of a muscle biopsy from an LOPD patient. Unstained LOPD muscle fibers were excited at 870 nm in 2-photon mode. SHG images (which reveal myosin bands, in green) and 2P-excited fluorescence images (which reveal autofluorescent particles, in red) were recorded simultaneously. In panels a & b myosin bands are weaker but appear uninterrupted around the particles. In panels c & d “black holes” characteristic of areas of autophagic debris are very clear (see [30]). Stacks of SHG and autofluorescence images of the fiber in panel d are shown in Fig. 4 of the main text. Bar: 20 μm. [file 2051-5960-2-2-S3.tiff]

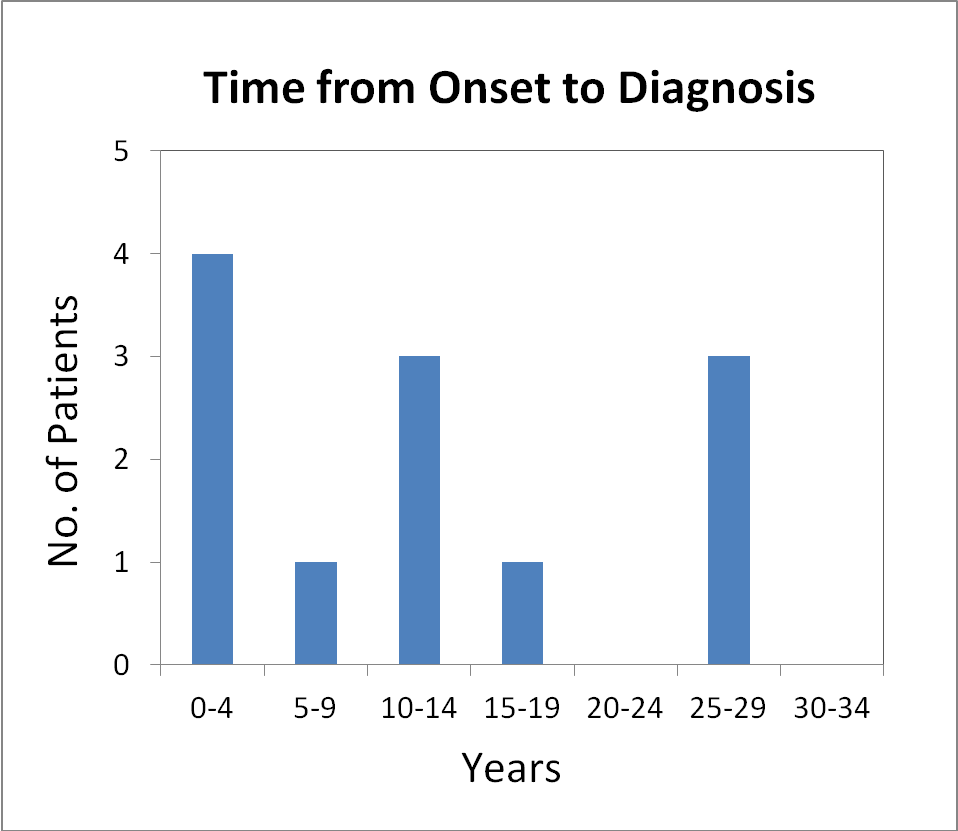

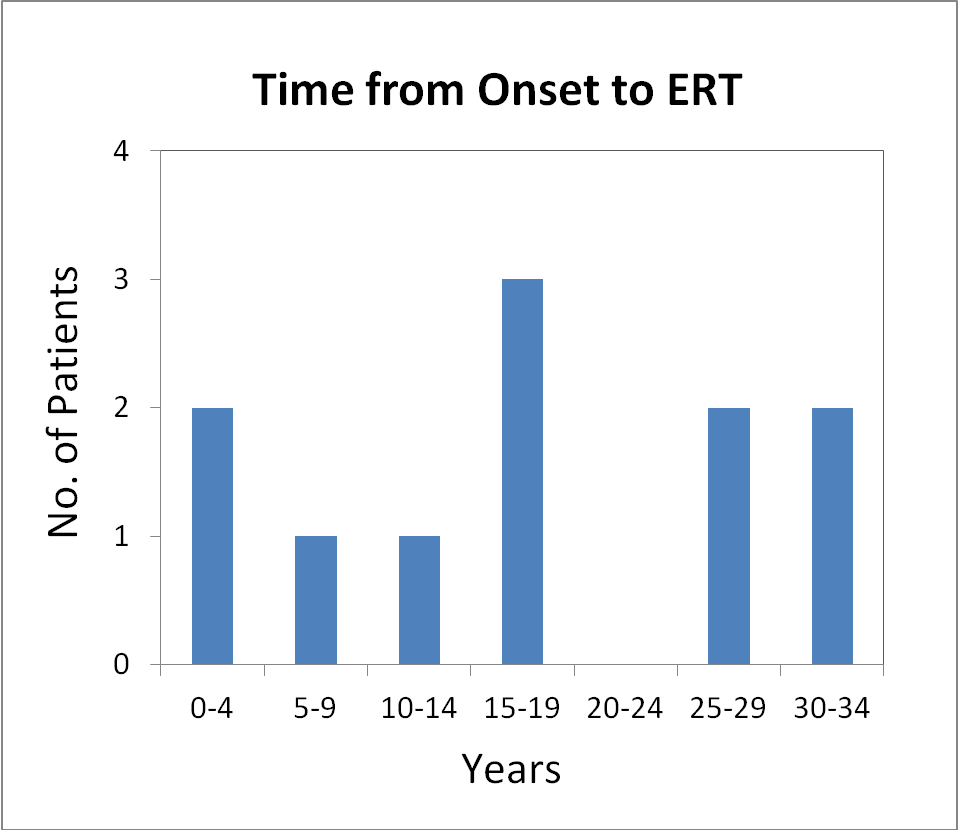

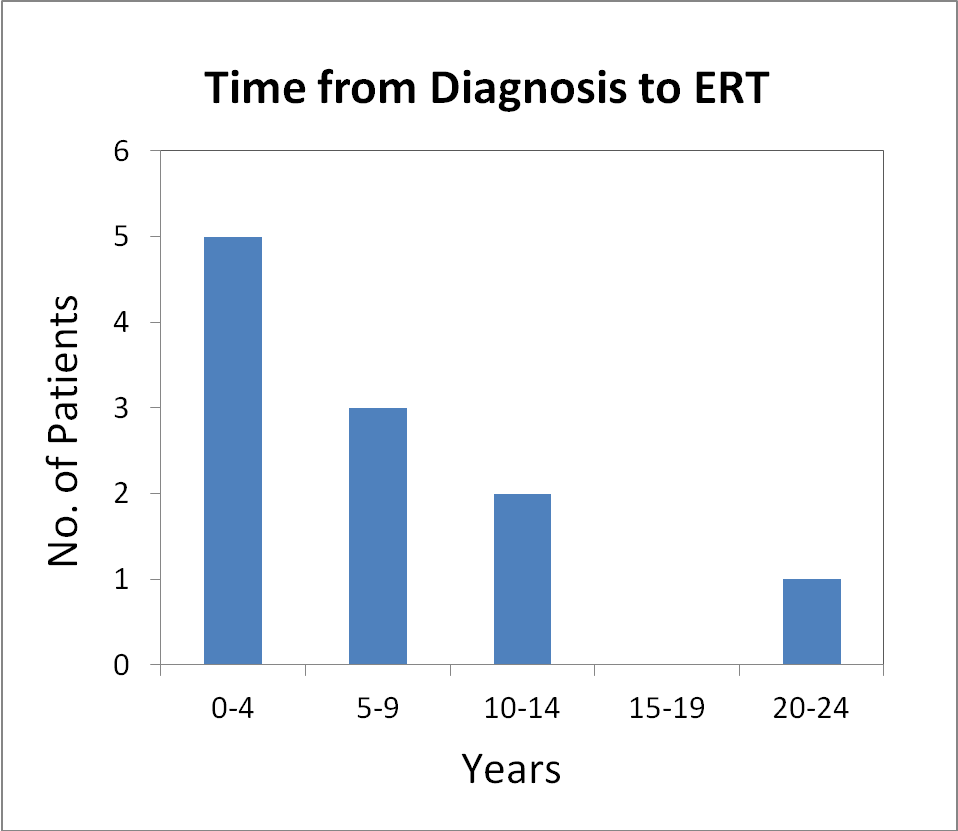
**Supplementary File 3. Time course of onset, diagnosis, and ERT initiation in adult-onset patients**

Supplement: Additional file 4: Figure S2 — Time course of onset, diagnosis, and ERT initiation in adult-onset patients. [file 2051-5960-2-2-S4.doc]

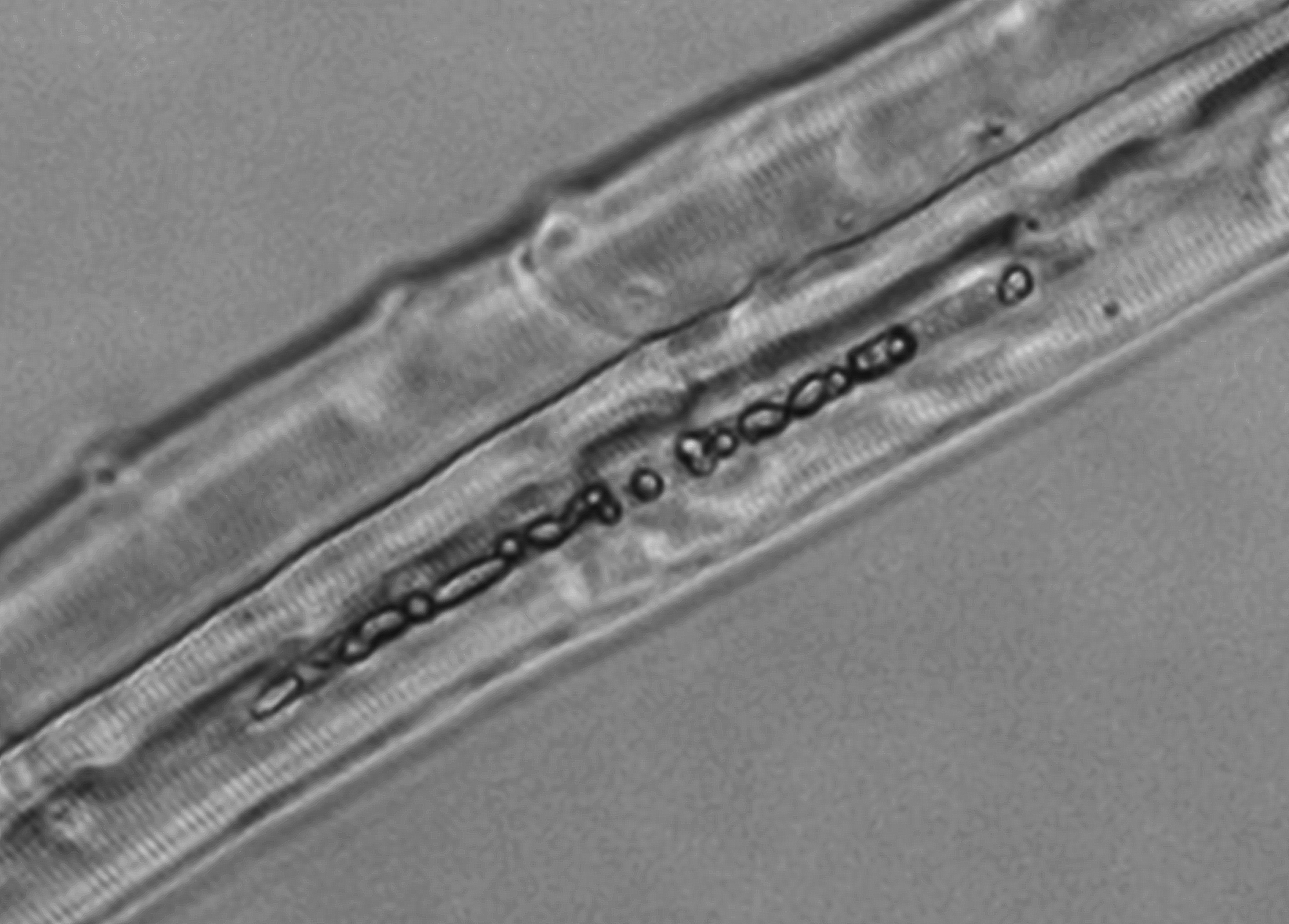

Supplement: Additional file 5: Figure S3 — Phase contrast image of muscle fibers from a muscle biopsy of patient HM1. The image, which shows large lipofuscin deposits in one of the two fibers, was taken by wide field microscopy. [file 2051-5960-2-2-S5.tiff]
